# The impact of China’s national essential medicine system on improving rational drug use in primary health care facilities: an empirical study in four provinces

**DOI:** 10.1186/s12913-014-0507-3

**Published:** 2014-10-25

**Authors:** Yan Song, Ying Bian, Max Petzold, Lingui Li, Aitian Yin

**Affiliations:** State Key Laboratory of Quality Research in Chinese Medicine, Institute of Chinese Medical Sciences, University of Macau, Av. Padre Tomás Pereira Taipa, Macau, China; Sahlgrenska Academy, University of Gothenburg, Box 100, S-405 30 Gothenburg, Sweden; College of Management, Ningxia Medical University, 1160 Shengli Street, Yinchuan, Ningxia Province China; Center for Health Management and Policy, Shandong University, 44 Wenhua Xilu, Jinan, Shandong Province China

**Keywords:** Rational drug use, Prescribing behavior, Essential medicines policy, Primary health care, China

## Abstract

**Background:**

The National Essential Medicine System (NEMS) is a new policy in China launched in 2009 to improve the appropriate use of medications. This study aims to examine the outcomes of the NEMS objectives in terms of the rational use of medicines in primary health care facilities in China.

**Methods:**

A total of 28,651 prescriptions were collected from 146 township health centers in four provinces of China by means of a field survey conducted in 2010–2011. Indicators of rational drug use were extracted and compared using a pre/post design and then evaluated with regard to the World Health Organization (WHO) Standard Guidelines and data from previous research.

**Results:**

The average number of drugs per prescription decreased from 3.64 to 3.46 (p < 0.01) between 2009 and 2010. Little effect was found for the NEMS on the average number of antibiotics per prescription, but the percentage of prescriptions including antibiotics decreased from 60.26 to 58.48% (p < 0.01). Prescriptions for injections or adrenal corticosteroids also decreased, to 40.31 and 11.16% of all prescriptions, respectively. All these positive issues were also recorded in 2011. However, each of the above values remained higher than WHO standards. The percentage of drugs prescribed from the Essential Drug List increased after the implementation of the NEMS (p < 0.01). Where the available data allowed changes in costs to be assessed, the average expense per prescription increased significantly, from 25.77 to 27.09 yuan (p < 0.01).

**Conclusions:**

The NEMS effectively improved rational medicine use in China. However, polypharmacy and the over-prescription of antibiotics and injections remain common. There is still a large unfinished agenda requiring policy improvements. Treatment guidelines, intensive support supervision, and continuing training for both professionals and consumers are the essential actions that need to be taken.

## Background

Rational drug use (RDU) requires that patients receive medications appropriate to their clinical needs, in doses that meet their own individual requirements, for an adequate period of time, and at the lowest cost to them and their community [1]. However, all countries, both rich and poor, have encountered problems with the insufficient use of beneficial, cost-effective drugs and the over use of unnecessary drugs. According to a World Health Organization (WHO) report, more than 50% of all medicines were prescribed, dispensed, or sold inappropriately worldwide, and 50% of patients failed to take their medicines correctly [2]. The medically inappropriate and economically inefficient use of drugs also occurs frequently in China. The consumption of intravenous fluids was estimated at eight bottles per person in China in 2009, a figure much higher than the overall global level (2.5–3.3 bottles). The overuse of antibiotics is another common form of medicine abuse in China. China is one of the heaviest users of antibiotics, with 70% of prescriptions containing antibiotics [3].

In China, hospitals can charge 15% over the wholesale price for drugs. This drug mark-up was initially introduced to compensate facilities for providing services below cost, as stipulated in the required fee schedule set by the government. However, this compensatory practice induced serious health hazards and encouraged physicians to over-prescribe unnecessary medicines, especially those that were more expensive or profitable. In 2009, the cost of drugs in China was 766 billion yuan, accounting for 41% of the total medical cost, and the cost of drugs per capita was 574 yuan [4]. The irrational use of medicines has resulted in not only a waste of resources but also adverse drug reactions (ADRs), drug resistance and increased drug-related morbidity and mortality [5]. It was estimated that about 2.5 million patients each year were admitted to the hospital owing to serious ADRs, and 60% of cases of acquired deafness in children were caused by irrational use of ototoxic drugs [6].

In 2009, increasing concerns regarding the appropriateness of medicinal drug use and access to safe and effective essential medicines led the Chinese government to introduce the National Essential Medicine System (NEMS) for public primary health care facilities, with the intention of extending it to private providers and hospitals. This program was a new policy in China and advocated prioritizing the use of essential medicines and the rational use of essential medicines. The program’s key objectives included removing the profit link between health institutions, doctors, and medicines and the improvement of medicine use. The core of the program was the Essential Drug List (EDL), which included selected low-cost medicines for common treatable diseases affecting vast numbers of people. The list for primary health care facilities contained 307 generic medicines (205 Western medicines and 102 traditional Chinese medicines). All public primary health care settings were required to stock and prescribe only drugs from the EDL and to sell them at cost (zero-profit drug policy). Clinical treatment guidelines and formulas for the administration of essential medicines were developed to regulate the behavior of health workers. Local social health insurance programs (including the New Rural Cooperative Medical Scheme) covered all essential medicines and provided a higher reimbursement for listed drugs than for non-listed drugs. However, different provinces varied in terms of the reimbursement rate.

To date, the initial three-year implementation plan has been realized. Clearly, there is a need to investigate the outcomes of the NEMS objectives with the further aim of contributing to the continued improvement of the system. Consequently, the purpose of this study was to examine the outcomes resulting from NEMS in terms of rational drug use in primary health care facilities and to provide up-to-date evidence and policy implications for the way forward.

## Methods

### Data collection

This was an ex post facto comparative study. Indicators of RDU in rural township health centers (THCs), a primary health care level in China, were compared before and after the implementation of the NEMS, and discussed with regard to WHO Standard Guidelines and data from other researches. Four provinces (Shandong, Zhejiang, Anhui, and Ningxia) representing contexts with varying levels of socioeconomic status (SES) were purposively selected as research areas. Shandong and Zhejiang are located in Eastern China and represent the developed parts of China. Anhui is part of the central region of China and an example of a moderately developed region. Ningxia is in the northwest part of China and is an undeveloped region. The New Rural Cooperative Medical Scheme covered over 95% of the rural population in each of the selected provinces until 2011 [7].

The survey in Zhejiang and Anhui was conducted in 2010. It was designed to reflect changes in the rational use of medicine between 2009 and 2010. Quota sampling was used to select two cities in each selected province (one with high SES and one with low SES). From each of the four selected cities, three counties were randomly chosen. All public THCs in the 12 chosen counties were investigated, resulting in a total of 113 THCs. In each THC, outpatient prescriptions from August 21 to August 30 in 2009 and 2010 were collected, and 200 prescriptions were selected using systematic random sampling (ten prescriptions per day). Generally, each consultation resulted in one prescription for drugs, treatments, or other recommendations.

The survey in Shandong and Ningxia was conducted in 2011. It was designed to reflect changes in the rational use of medicine during 2009–2011. A county of medium size and middle development level in Shandong was selected for sampling. All 17 THCs in this county were investigated. In Ningxia, 16 THCs were selected with quota sampling, drawing upon divisions previously made by local officials dividing the province into low, middle, and high SES levels. One county was randomly selected from each of the three SES strata. The counties selected were Xiji, Tongxin and Qingtongxia. Based on size of the selected county, eight THCs were randomly selected from Xiji, and four were randomly chosen from each of the other two selected counties. In each THC, outpatient prescriptions issued between January 1 and June 30 in 2009, 2010, and 2011 were collected, and a sample of 180 prescriptions was drawn using systematic random sampling (at least ten prescriptions per month). The sampling method is different in the two surveys. During the first stage of research, in Zhejiang and Anhui, we encountered a few unexpected problems in carrying out the sampling plan. Based on this experience, we made some adjustments in the sampling in Shandong and Ningxia.

All parts of the present study were approved by the ethics committee of Institute of Chinese Medical Sciences at the University of Macau. Data collection was anonymous (name or identifying information of the patients, general practitioners, and pharmacists were not obtained). The final data available for analysis in this study consist of 28,651 prescriptions, described in Table 1.

**Table 1:** **Numbers of township health centers and prescriptions selected in four Chinese provinces**

	**THCs**		**Prescriptions**	
		**2009**	**2010**	**2011**
Anhui	26	2575	2570	0
Zhejiang	87	8408	8700	0
Shandong	17	1046	1009	982
Ningxia	16	1033	1219	1109
Total	146	13062	13498	2091

Note: THCs = township health centers.

### Indicators

Drug use indicators for primary health care facilities from the WHO/International Network for the Rational Use of Drugs have a central place in assessing drug use practices, as they have been shown to be both feasibly measured and informative as first level indicators [8,9]. The final versions of the pretested indicators in this study were derived by taking these indicators as a reference and combining them with information about the specific situation in China. Six indicators were pretested in this study:

1. Average number of drugs per prescription (ANDPP)
2. Percentage of drugs prescribed from the EDL (PED)
3. Average number of antibiotics per prescription (ANAPP)
4. Percentage of prescriptions including an antibiotic (PPA)
5. Percentage of prescriptions including an injection (PPI)
6. Percentage of prescriptions including adrenal corticosteroid (PPC)

### Data analysis

We extracted the data from each prescription, including number of medicines, number of essential medicines, number of antibiotics, use of injections, and use of adrenal corticosteroids. In Shandong and Ningxia, the total medical cost per prescription was also accessible. The drug use indicators were computed as follows:

$$ \mathrm{ANDPP}=\frac{\mathrm{total}\ \mathrm{number}\ \mathrm{of}\ \mathrm{drugs}\ \mathrm{prescribed}}{\mathrm{total}\ \mathrm{number}\ \mathrm{of}\ \mathrm{prescriptions}\ \mathrm{surveyed}} $$

$$ \mathrm{PED}=\frac{\mathrm{total}\ \mathrm{number}\ \mathrm{of}\ \mathrm{essential}\ \mathrm{medicines}\ \mathrm{prescribed} \times 100\%}{\mathrm{total}\ \mathrm{number}\ \mathrm{of}\ \mathrm{drugs}\ \mathrm{prescribed}} $$

$$ \mathrm{ANAPP}=\frac{\mathrm{total}\ \mathrm{number}\ \mathrm{of}\ \mathrm{antibiotics}\ \mathrm{prescribed}}{\mathrm{total}\ \mathrm{number}\ \mathrm{of}\ \mathrm{prescriptions}\ \mathrm{surveyed}} $$

$$ \mathrm{P}\mathrm{P}\mathrm{A} = \frac{\mathrm{number}\ \mathrm{of}\ \mathrm{prescripitons}\ \mathrm{including}\ \mathrm{an}\ \mathrm{an}\mathrm{tibiotic} \times 100\%}{\mathrm{total}\ \mathrm{number}\ \mathrm{of}\ \mathrm{prescriptions}\ \mathrm{surveyed}} $$

$$ \mathrm{P}\mathrm{P}\mathrm{I} = \frac{\mathrm{number}\ \mathrm{of}\ \mathrm{prescriptions}\ \mathrm{including}\ \mathrm{an}\ \mathrm{injection} \times 100\%}{\mathrm{total}\ \mathrm{number}\ \mathrm{of}\ \mathrm{prescriptions}\ \mathrm{surveyed}} $$

$$ \mathrm{P}\mathrm{P}\mathrm{C} = \frac{\mathrm{number}\ \mathrm{of}\ \mathrm{prescriptions}\ \mathrm{including}\ \mathrm{adrenal}\ \mathrm{corticosteroid} \times 100\%}{\mathrm{total}\ \mathrm{number}\ \mathrm{of}\ \mathrm{prescriptions}\ \mathrm{surveyed}} $$

Data analyses were performed with IBM SPSS Statistics Version 19.0 (Armonk, New York, USA). The 2009 data were considered to be in the pre-reform period, and the 2010 and 2011 data were considered to be in the post-reform period. Descriptive analyses of variables were carried out on the basis of means and corresponding percentages. Student’s t-test was performed to compare the means, and Fisher’s exact test was used to compare the percentage distributions between the two periods. Additionally, during the analysis, the consumer price index was used to correct the total medical cost per prescription for inflation or deflation between the survey year and the base year (2009), and the values of this variable were transformed into natural logarithms of the observed value to address the positive skew of the expenditure data.

## Results

### General situation

Table 2 shows the indicators and corresponding changes in the use of essential medicines, antibiotics, injections, and adrenal corticosteroids for outpatient visits in 146 facilities from the four selected provinces between 2009 and 2010.

**Table 2:** **Drug use indicators at 146 township health centers in four Chinese provinces, 2009–2010**

	**Anhui**			**Zhejiang**			**Shandong**			**Ningxia**			**Total**		
	**2009**	**2010**		**2009**	**2010**		**2009**	**2010**		**2009**	**2010**		**2009**	**2010**	
ANDPP	4.17	4.25	↑	3.57	3.3	↓*	3.37	3.28	↓	3.23	3.12	↓	3.64	3.46	↓*
PED, %	75.62	86.88	↑*	61.31	83.29	↑*	50.65	49.41	↓	55.35	60.59	↑	63.33	79.89	↑*
ANAPP	0.96	1	↑	0.79	0.75	↓*	0.73	0.71	↓*	0.72	0.81	↑*	0.81	0.8	↓
PPA, %	67.81	70.35	↑*	59.92	55.52	↓*	51.43	51.54	↑	53.15	60.38	↑*	60.26	58.48	↓*
PPI, %	51.11	50.43	↓	43.74	40.35	↓*	30.59	29.04	↓	28.36	27.97	↓	42.93	40.31	↓*
PPC, %	22.68	22.10	↓	11.91	9.22	↓*	11.09	10.70	↓	1.65	2.30	↑	13.15	11.16	↓*

Note: ↑ = 2010 − 2009 > 0; ↓ = 2010 − 2009 < 0.

ANDPP = average number of drugs per prescription; PED = percentage of drugs prescribed from the Essential Drug List; ANAPP = average number of antibiotics per prescription; PPA = percentage of prescriptions including an antibiotic; PPI = percentage of prescriptions including an injection; PPC = percentage of prescriptions including adrenal corticosteroid.

*= p < 0.05, p-value refers to t-test to compare means or exact test to compare proportion distributions between the two periods.

The mean number of drugs prescribed per prescription decreased from 3.64 to 3.46 between 2009 and 2010. Significantly fewer drugs were prescribed per prescription after the NEMS (p < 0.01). In 2010, 15.06% of outpatient prescriptions included one drug, 23.02% included two drugs, 20.98% included three drugs, and 40.91% included four or more (Table 3). The prescriptions where four or more drugs were prescribed decreased compared with 2009 (p < 0.05).

**Table 3:** **Prescriptions by number of drugs per prescription, 2009 and 2010**

**No. of drugs per prescription**	**Prescriptions, N (%)** ^**Δ**^		**RD (95% CI)**
	**2009**	**2010**	
0	2 (0.02)	4 (0.03)	+0.01 (−0.02, +0.05)
1	1745 (13.36)	2036 (15.06)	+1.70 (+0.87, +2.54)
2	2653 (20.31)	3111 (23.02)	+2.71 (+1.72, +3.70)
3	2657 (20.34)	2836 (20.98)	+0.64 (−0.33, +1.61)
4	2028 (15.53)	1847 (13.67)	−1.86 (−2.71, −1.01)
5	1796 (13.75)	1660 (12.28)	−1.47 (−2.28, −0.66)
≥ 6	2181 (16.70)	2022 (14.96)	−1.74 (−2.62, −0.86)

Note: RD = the difference of proportion between the years; CI=confidence interval.

^Δ^= There is statistically significant difference (p < 0.05) in proportion distributions of prescriptions by number of drugs between 2009 and 2010.

Moreover, overall, 79.89% of drugs prescribed were from the EDL in 2010, compared with 63.33% in 2009. Of all outpatient prescriptions in 2009, 60.26% included one or more antibiotics. This percentage decreased to 58.48% in 2010 (p < 0.01). The mean number of antibiotics per prescription was 0.8 in 2010, almost equal to the mean of 0.81 in 2009. However, the number of antibiotics per prescription decreased in Zhejiang and Shandong. The absence of an overall reduction was entirely because of more antibiotics per prescription in Anhui and Ningxia. Additionally, in 2009, 2.08% of all prescriptions contained three or more antibiotics; this proportion increased to 2.26% in 2010 (p < 0.05) (see Table 4).

**Table 4:** **Prescriptions by number of antibiotics per prescription, 2009 and 2010**

**No. of antibiotics per prescription**	**Prescriptions, N (%)** ^**Δ**^		**RD (95% CI)**
	**2009**	**2010**	
No antibiotics	5191(39.74)	5612(41.52)	+1.78 (+0.60, +2.96)
With antibiotics	7871(60.26)	7904(58.48)	−1.78 (−2.96, −0.60)
1	5450(41.72)	5403(39.97)	−1.75 (−3.28, −0.22)
2	2149(16.45)	2195(16.24)	−0.21 (−1.36, +0.94)
**≥**3	272(2.08)	306(2.26)	+0.18 (−0.27, +0.63)

Note: RD = the difference of proportion between the years; CI=confidence interval.

^Δ^= There is statistically significant difference (p < 0.05) in proportion distributions of prescriptions by number of antibiotics between 2009 and 2010.

The rates of prescriptions containing injections decreased in all selected provinces between 2009 and 2010. On average, 40.31% of consultations resulted in an injection in 2010. The use of adrenal corticosteroids was also improved significantly. The percentage of prescriptions that included adrenal corticosteroids decreased to 11.16% in 2010.

### Further investigation in Shandong and Ningxia

The situation of rational drug use in 2011 was further investigated in Shandong and Ningxia. As is shown in Table 5, results of this additional investigation revealed that the rate of medicines prescribed from the EDL increased each year after the implementation of the NEMS (p < 0.01). There were significantly fewer prescriptions with an antibiotic in 2011, and the average number of antibiotics per prescription was also significantly lower (p < 0.01). Additionally, the analysis indicated that fewer outpatient prescriptions included injections (p < 0.01). Frequently-prescribed injections (excluding traditional Chinese medicine injections) included solutions correcting water, electrolyte, and acid–base disturbances (35.21%); antibiotics (23.12%); vitamins and minerals (9.66%); antiviral medicines (7.33%); and adrenal corticosteroids (5.08%). However, the available data do not demonstrate a clear improvement in ANDPP or PPC in 2011, compared with 2009.

**Table 5:** **Drug use indicators at 33 township health centers in Shandong and Ningxia, China, 2009–2011**

	**Shandong**			**Ningxia**			**Total**		
	**2009**	**2010**	**2011**	**2009**	**2010**	**2011**	**2009**	**2010**	**2011**
ANDPP	3.37	3.28	3.36	3.23	3.12	3.15	3.30	3.19	3.25
PED, %	50.65	49.41	65.67	55.35	60.59	70.64	53.03	55.49	68.31
ANAPP	0.73	0.71	0.66	0.72	0.81	0.70	0.73	0.77	0.68
PPA, %	51.43	51.54	47.15	53.15	60.38	54.19	52.28	56.37	50.88
PPI, %	30.59	29.04	26.68	28.36	27.97	22.36	29.50	28.50	24.40
PPC, %	11.09	10.70	11.20	1.65	2.30	1.89	6.40	6.10	6.30

Note: ANDPP = average number of drugs per prescription; PED = percentage of drugs prescribed from the Essential Drug List; ANAPP = average number of antibiotics per prescription; PPA = percentage of prescriptions including an antibiotic; PPI = percentage of prescriptions including an injection; PPC = percentage of prescriptions including adrenal corticosteroid.

The total medical cost per prescription significantly increased between 2009 and 2011, from 25.77 to 27.09 yuan (p < 0.01). Furthermore, the cost of prescriptions containing antibiotics were higher than the cost of those without antibiotics (p < 0.01). Similarly, the cost of prescriptions where an injection was prescribed was significantly higher than that where no injection was prescribed (p < 0.01).

## Discussion

The township health center plays an important role in providing primary health care services to the large population in rural China. The findings of this study may provide quantitative baseline data for monitoring and improving prescribing processes and rational drug use in China.

First, our study revealed that significantly fewer drugs were prescribed per prescription after China’s implementation of the NEMS (p < 0.01). In 2010, patients were prescribed an average of 3.46 drugs per prescription, which was however still double the derived WHO standard value of 1.6–1.8 [10]. It was also higher than the average of 2.2 drugs prescribed per patient visit found in a study of 17 developing countries, where the highest national average was 3.8, found in Indonesia and Nigeria [11]. Thus, our study indicates that the trend towards polypharmacy persisting in China was among the world’s most severe cases. Furthermore, we found that about 27.24% prescriptions contained five or more different kinds of drugs in 2010; the highest number was 32 drugs for a single prescription. Multi-drug use may increase the risk of undesirable drug interactions and needs more attention.

Following the implementation of the NEMS, all THCs were required to stock and prescribe drugs solely from the EDL and to sell them at cost, a policy aimed at ending the interest link between physicians and drugs and reducing incentives for health care providers to over-prescribe. The current trend towards polypharmacy may be attributed to patients’ demands; patients believe that prescribing more drugs will ensure improvement and facilitate the cure of their conditions more quickly [12]. Another contributing factor could be that the treatment is based purely on symptoms instead of a full diagnosis. Though clinical treatment guidelines and formulas for essential medicines have been developed, they have not yet been fully accepted or followed by physicians in THCs. Another possible factor could be that “bargain mentality” encourages irrational use. For example, in Ningxia, there is an annual medical compensation for outpatient services (about 30 yuan per person). Patients prefer to get medicines for free and always pressure prescribers to prescribe medicines within the quota, especially at the end of the fiscal year, regardless of whether they need these drugs or not. Thus, RDU education for both health care providers and consumers should be further promoted.

Second, analysis of the data revealed that essential medicines constitute a reasonably high proportion of all medicines prescribed in THCs after the implementation of the NEMS. On average, 80% of the drugs prescribed appeared on the EDL in 2010. The priority use of essential medicines is worth advocating. However, doctors in primary health care facilities should not be limited to prescribing only essential medicines and should have some scope for autonomy within a certain range. It is rare for a country to reduce medicine costs and promote rational medication by controlling the prescription practices of doctors with an EDL covering a few medicine categories. This rarity is because of the specificity of the medical industry and the individual variations within it across contexts [13].

Third, our study showed that the number of prescriptions where one or more antibiotics were prescribed decreased to 58.48% in 2010 (p < 0.01), whereas the WHO recommendation is 20.0–26.8% [8]. Moreover, the proportion containing more antibiotics increased somewhat, and the mean number of antibiotics per prescription has not changed. Antibiotics are essential, but their overuse can increase antibiotic resistance, which will endanger their therapeutic effectiveness, increase treatment failure, and, as a result, lead to longer and more severe illness episodes associated with higher costs and an increased mortality rate [14]. The major factors reported to influence the high percentage of antibiotics prescribed elsewhere are physicians’ lack of knowledge about appropriate antibiotic use, including the overestimation of the severity of the illness to justify prescribing an antibiotic, and pressure from patients who believe that antibiotics provide rapid symptomatic relief from disease [15,16]. The same factors may play a role in China. Hence, follow-up research should focus on the improvement of the use and management of antibiotics. There is also an urgent need for public education.

Fourth, our results showed that the use of injections and adrenal corticosteroids improved significantly after the implementation of the NEMS. However, the overall percentage of prescriptions containing injections in Shandong and Ningxia in 2011 was 24.4%, still higher than the percentage reported previously in 35 developing countries (22.8%) [17]. In China, the mistaken belief that “Injections mean you will get well sooner” has always caused patients to request injections [18]. Compared with oral drug therapy, injections are more expensive because of the additional cost of syringes, sterilization control, and well-trained personnel. In addition to increasing the economic burden of patients, overuse of injections brings risks of unsafe needles that can increase the transmission of AIDS, hepatitis B and C, and other blood-borne diseases [19]. Most notably in our survey, 23.12% of injections prescribed were antibiotics, potentially increasing the risk of antibiotic resistance.

Finally, the cost analysis in Shandong and Ningxia revealed that the total medical cost per prescription significantly increased between 2009 and 2011. With the price control policy of the NEMS, medicine prices in 2011 were reduced by 25% from 2009 prices [20]. However, medical costs did not fall correspondingly. The poor outcome might be partly caused by patients being treated with too many medicines, especially antibiotics. The results confirmed that the medical cost per prescription was higher with antibiotics or injections than that without, a finding that is consistent with other research [21]. Furthermore, a report from three Chinese provinces previously revealed that certain manufacturers ceased production of some essential drugs because the price controls and recommended procurement procedures in the NEMS severely curtailed their profits. The report also revealed that many health care providers complained that the prices they were paying for some drugs under the NEMS were higher than those they had paid before the NEMS was introduced in their facility [22]. These factors could be a contributor to rising medical costs. Additionally, the financial incentives that manufacturers provide to hospitals and doctors might also lead to the frequent use of medicines with higher prices due to the persistence of widespread corruption in the procurement process [23–25]. Where profits from selling drugs have been eliminated, appropriate financial compensation mechanisms are needed to ensure the sustainable development of the primary health care facilities, because drug revenue previously accounted for more than 50% of hospital revenue in China [26].

The results of this study should be interpreted in light of several limitations. First, China’s NEMS has recently been launched, and we collected post-implementation information soon after the introduction. Its effectiveness and impacts have not been fully realized, and there is limited empirical evidence to draw upon in making conclusions about its efficacy. Second, the results of this study were based only on four selected provinces. Although these four provinces were selected purposively to explore different development levels, the conclusions of this study should be generalized to the whole of China only with caution. Third, the prescription analysis conducted in this study only examined outpatient prescriptions and excluded inpatient prescriptions mainly owing to limited data availability.

## Conclusions

In conclusion, most of the observed indicators in 2010 and 2011 have improved compared with 2009. There was a positive association between the NEMS and the more appropriate use of medications in primary health care settings in China. However, polypharmacy and the over-prescription of antibiotics and injections remain common. At the time of our data collection, the RDU indicators had not yet met the WHO Standard Guidelines. The NEMS agenda remains largely unfinished, and its realization requires further policy improvements. In light of this need for future improvements, we provide some recommendations based on the above findings and discussion. First, treatment guidelines should be highlighted and closely followed. Health delivery systems should routinely assess medicine use to encourage rational prescription. Next, the concept of rational drug use should be incorporated into the basic and continuing education of health care providers. Finally, it is necessary to improve training on RDU for consumers to gradually change their mistaken beliefs and thus reduce their demand for drugs that do not bring additional health benefits.

## Consent

Written informed consent was obtained from the patient for the publication of this report and any accompanying images.

## Abbreviations

RDU: Rational Drug Use
NEMS: National Essential Medicine System
EDL: Essential Drug List
THC: Township Health Center
ADR: Adverse Drug Reaction
WHO: World Health Organization
ANDPP: Average number of drugs per prescription
PED: Percentage of drugs prescribed from the Essential Drug List
ANAPP: Average number of antibiotics per prescription
PPA: Percentage of prescriptions including an antibiotic
PPI: Percentage of prescriptions including an injection
PPC: Percentage of prescriptions including adrenal corticosteroid
